# Contribution of Chronic Fatigue to Psychosocial Status and Quality of Life in Spanish Women Diagnosed with Endometriosis

**DOI:** 10.3390/ijerph17113831

**Published:** 2020-05-28

**Authors:** Antonio Mundo-López, Olga Ocón-Hernández, Ainhoa P. San-Sebastián, Noelia Galiano-Castillo, Olga Rodríguez-Pérez, María S. Arroyo-Luque, Manuel Arroyo-Morales, Irene Cantarero-Villanueva, Carolina Fernández-Lao, Francisco Artacho-Cordón

**Affiliations:** 1 Department of Radiology and Physical Medicine, University of Granada, E-18016 Granada, Spain; antonio@alarconpsicologos.com (A.M.-L.); ainhoassp@correo.ugr.es (A.P.S.-S.); olgarope@correo.ugr.es (O.R.-P.); marisi_7@hotmail.com (M.S.A.-L.); 2 Clinic Psychology Center Alarcón (CPCA), E-18004 Granada, Spain; 3 Biohealth Research Institute in Granada (ibs.GRANADA), E-18012 Granada, Spain; ooconh@ugr.es (O.O.-H.); noeliagaliano@ugr.es (N.G.-C.); marroyo@ugr.es (M.A.-M.); irenecantarero@ugr.es (I.C.-V.); 4 Gynaecology and Obstetrics Unit, ‘San Cecilio’ University Hospital, E-18016 Granada, Spain; 5 Department of Physiotherapy, University of Granada, E-18016 Granada, Spain; 6 “Cuídate” Support Unit for Oncology Patients (UAPO), Sport and Health University Research Institute (iMUDS), E-18016 Granada, Spain; 7 CIBER Epidemiology and Public Health (CIBERESP), E-28029 Madrid, Spain

**Keywords:** chronic fatigue, endometriosis, psychosocial status, quality of life

## Abstract

Aim: To analyze the levels of chronic fatigue in Spanish women with endometriosis and its relationship with their psychosocial status and quality of life (QoL). Methods: A total of 230 Spanish women with a clinical diagnosis of endometriosis were recruited. Chronic fatigue (Piper Fatigue Scale) and pelvic pain (Numeric Rating Scale) were evaluated. An on-line battery of validated scales was used to assess psychosocial status [Hospital Anxiety and Depression Scale, Scale for Mood Assessment, Pain Catastrophizing Scale, Pittsburgh Sleep Quality Index, Gastrointestinal Quality of Life Index, Female Sexual Function Index and Medical Outcomes Study-Social Support Survey] and QoL [Endometriosis-Health Profile questionnaire-30]. Associations between fatigue and both psychosocial and QoL outcomes were explored through multivariate regression models. Results: One-third and one-half of women showed moderate and severe fatigue, respectively. Fatigue was associated with higher anxiety and depression, poorer sleep quality, poorer sexual functioning, worse gastrointestinal health, higher catastrophizing thoughts, higher anger/hostility scores and lower QoL (*p*-values < 0.050). Moreover, fatigue and catastrophizing thoughts showed a mediating effect on the association between pelvic pain and QoL. Conclusion: This work reveals the important role of fatigue in the association between pain, psychosocial status, and QoL of Spanish women with endometriosis.

## 1. Introduction

Endometriosis, characterized by the ectopic development of endometrial-like tissue, is among the most commonly diagnosed benign diseases in women of reproductive age [1]. Diagnostic delay and the fact that diagnosis is often overlooked by primary care doctors make the prevalence of the disease difficult to establish. Nevertheless, prevalence estimates range from 1–2% when considering populations at low risk to 10% when high-risk populations are considered [2]. However, despite the benign nature of this disease, huge direct and indirect costs (raising up to more than $12,000 – $15,000 in some countries) have been evidenced to be associated with endometriosis [3].

Pain in the pelvic region is acknowledged to be the most characteristic symptom of women with endometriosis, which is intensified during the menstruation period (dysmenorrhea) and during the performance of daily activities such as defecation (dyschezia), urination (dysuria) or sexual relationships (dyspareunia) [4,5]. The contribution of pelvic pain (PP) to the psychosocial status and the quality of life (QoL) of women with endometriosis has been extensively addressed [6,7,8,9,10]. Additionally, chronic fatigue, i.e., the perception of physical tiredness and lack of energy distinct from sadness or weakness, is another endometriosis-related symptom, as recently identified in women with endometriosis [11]. However, the role of chronic fatigue on patients’ lives has been poorly addressed, although a few qualitative studies have indicated that affected women ascribed social and work impairments to fatigue [12,13]. Contrary, there is consistent evidence of the relevant role of fatigue in different subsets of patients experiencing chronic pain, suggesting that fatigue hinders the completion of routines and significant activities, and therefore, severely reduces QoL [14,15].

Moreover, the relevant contribution of chronic fatigue to the presence of psychosocial impairments in patients with autoimmune diseases [16] or neurological problems [17] has been reported. However, contrary to the well-established relationship between chronic pain, psychosocial problems and QoL in women with endometriosis, there is a scarcity of published studies addressing the contribution of chronic fatigue to the symptomatic burden in women with endometriosis under medical treatment. Thus, the aim of this study was to explore the presence of chronic fatigue in Spanish women diagnosed with endometriosis and its contribution to the psychosocial status and QoL.

## 2. Material and Methods

### 2.1. Study Population

A total of 230 women with a clinical diagnosis of endometriosis, from different regions of Spain, were enrolled in this cross-sectional study from January to July 2019. Recruitment of women was carried out in combination with both gynecologists and Spanish associations of endometriosis patients, which advertised the study in their social networks. The inclusion criteria were: to have attended a gynecological visit with any participating gynecologist or to belong to any of the Spanish associations of endometriosis patients; to be diagnosed with endometriosis (either by laparoscopy, magnetic resonance or ultrasound imaging, or based on symptoms); and to have the ability and availability to use an electronic device with internet connection (computer, tablet or mobile phone). The exclusion criteria were: to live in another country or to be a non-Spanish speaker. The survey was designed to consider researchers’, gynecologists’ and patients’ opinion about the most relevant aspects that should be addressed. Interested women received a link for the completion of an on-line questionnaire. Prior to this, women were informed about the nature and objectives of the study, and they were requested to read and sign the informed consent. No personal information was asked in the questionnaire, and data was extracted to create an anonymized database. This study was carried out following the principles of the Declaration of Helsinki and Biomedical Research Law 14/2007 and was approved by the Research Ethics Committee of Granada (1733-N-18).

### 2.2. Assessment of Self-Reported Intensity of Chronic Fatigue and Pain

Chronic fatigue was assessed through the Spanish version of the Piper Fatigue Scale (PFS) [18,19]. PFS is a validated 22-item tool for self-reported chronic fatigue in breast cancer survivors, but also in patients with gynecological disorders [20] or coronary diseases [21]. It includes four dimensions of subjective fatigue: “behavioral/severity”, “affective meaning”, “sensory” and “cognitive/mood”. Scores range from 0 to 10, with higher scores indicating greater fatigue. It has demonstrated high reliability and validity (Cronbach’s alpha 0.86). Participants were divided into three groups according to the clinically significant fatigue criteria: mild (≤ 4.0), moderate (4–7) or fatigued (≥ 7), according to the value obtained for the PFS total score [18,22].

Intensity of self-reported PP during the last week was assessed through a numeric rating scale (NRS). This 11-point Likert scale (0 = no pain; 10 = unbearable pain) is one of the best single-item methods available to estimate the intensity of pain [23,24,25]. Pain severity was categorized as mild (0–3), moderate (4–7) and severe (8–10), as reported elsewhere [26].

### 2.3. Psychosocial Assessment

Participants were asked to complete the Hospital Anxiety and Depression Scale (HADS), the Scale for Mood Assessment (EVEA), the Pain Catastrophizing Scale (PCS), the Pittsburgh Sleep Quality Index (PSQI), the Female Sexual Function Index (FSFI), the Gastrointestinal Quality of Life Index (GIQLI) and the Medical Outcomes Study-Social Support Survey (MOS-SSS).

Anxiety and depression were assessed through the Spanish version of the HADS [27], a self-assessment mood scale validated for its use in non-psychiatric hospital outpatients to determine their levels of anxiety and depression [28]. It has two subscales (anxiety and depression), each ranging from 0 to 21, showing adequate reliability (Cronbach’s alpha 0.86) [29]. Higher scores on the subscale indicate higher degrees of anxiety and depression [30]. For both subscales, available cut-off scores allowed the identification of non-cases (≤7) mild (8–10), moderate (11–14) and severe cases (15–21) [28,31]. Additionally, the Spanish version of the EVEA scale was partially used to evaluate “anger/hostility” and “happiness” through the corresponding subscales. Item scores are evaluated with Likert scales ranging from 0 to 10, and the values per category are obtained from mean scores. EVEA subscales have shown good reliability (Cronbach’s alpha range between 0.88 and 0.93) [32].

Catastrophic thoughts about pain were assessed through the Spanish version of the PCS, a 13-item, validated, self-report instrument with adequate reliability (Cronbach’s alpha 0.79) [33]. This measure has a 5-point Likert-style response scale and the scoring range is 0–52, with higher scores indicating higher levels of catastrophic thoughts. Previous studies have shown a cut-off of more than 30 points to be associated with clinical relevance [34].

Sleep quality was assessed using the Spanish validated version of the PSQI [35]. The PSQI is a 19-item, validated, self-report scale used to measure quality and patterns of sleep, with adequate reliability (Cronbach’s alpha 0.87). Scores range from 0 to 21, with higher scores representing poorer sleep quality [36]. It has been proposed that a total score ≤5 indicates good sleep quality while a total score > 5 indicates poor sleep quality [36].

Sexual function was assessed through the Spanish version of the FSFI [37]. This is a 19-item questionnaire, validated, multidimensional self-report instrument for assessing the major aspects of female sexual dysfunction and sexual satisfaction [37,38]. The FSFI score ranges from 0 to 36. Higher scores represent better sexual function, considering that patients with a FSFI total score below 26 are categorized as sexual dysfunctional, whereas those scoring at or above this cut-off score are categorized as sexually functional [39]. Adequate reliability has been reported (Cronbach’s alpha >0.70 for all domains).

Digestive complaints were assessed through the Spanish version of the GIQLI [40], a self-administered 36-item questionnaire concerning digestive symptoms, physical status, emotions, social dysfunction and effects of medical treatment. Each item scores from 0 to 4 with the total score ranging from 0 to 144, higher scores representing better quality of life. GIQLI also measures physical well-being, mental well-being, digestion and defecation [41].

The Spanish version of the MOS-SSS scale was used to assess the extent to which the person has the support of others to face stressful situations [42]. It is comprised of 19 items with a 5-point Likert-style response, with higher scores representing better social support. This measure has shown good psychometric quality in different studies using diverse populations and clinical scenarios (Cronbach’s alpha 0.94) [43].

### 2.4. Quality of Life

The Spanish version of the validated Endometriosis Health Profile-30 (EHP-30) questionnaire was used for the assessment of QoL in participating women [44]. This 30-item scale has five subscale scores: pain, control and powerlessness, social support, emotional well-being and self-image. Each subscale is standardized on a scale of 0–100, where 0 indicates the best health status and 100 the worst health status. Scale scores for each scale are calculated from the total of the raw scores of each item in the scale divided by the maximum possible raw score of all the items in the scale, multiplied by 100. This instrument has shown good internal consistency reliability, with Cronbach’s alpha >0.88 for all subscales.

### 2.5. Statistical Analysis

The sociodemographic and gynecological characteristics of participants and scores for PP and chronic fatigue were expressed as geometric means (GMs) with geometric standard deviation (GSD), or as percentages, depending on the continuous or categorical nature of the variable. Scores for QoL, i.e., psychosocial outcomes, were expressed as GM with GSD, as minimum and maximum values, and as percentiles (25, 50, and 75). When clinical cut offs were available, variables were categorized and expressed as percentages.

To improve normality of the data, psychosocial outcomes were log-transformed and, therefore, β coefficients are also presented as exp(β). Associations between fatigue severity (mild/moderate/severe), psychosocial outcomes and QoL were assessed by using linear regression models adjusted for sociodemographic and gynecological characteristics, including age, schooling, civil status, severity of premenstrual syndrome (none, mild, moderate or severe), type of diagnosis, time since diagnosis and number of surgeries. Additional models adjusted for severity of last week PP are also presented. Moreover, the mediation effect of fatigue and pain catastrophizing thoughts on the relationship between last week PP intensity and QoL was assessed through the macro PROCESS for Statistical Package for the Social Sciences (SPSS) [45], and mediating effects were considered significant when zero was not located within the confidence intervals.

The statistical significance level was set at *p* = 0.05. Analyses were performed using SPSS v23.0 statistical software (IBM, Chicago, IL, USA), while figures were designed with Graphad Prism 5.0 software (San Diego, CA, USA). The post-hoc analysis to estimate the power (1-β) of the statistical analysis was conducted using G*Power 3.1.9.7 statistical software (Düsseldorf University, Düsseldorf, Germany). For the main analysis between chronic fatigue and QoL, it revealed that, for an R^2^ of 0.28 assuming an α-error of 0.05, the power was >0.99.

## 3. Results

A total of 241 women were interested in the study. However, 11 (4.6%) women did not meet inclusion/exclusion criteria. Finally, 230 women agreed to participate. Baseline characteristics of the study population are summarized in Table 1. The mean (±SD) age of the study population was 36.7 ± 5.2 years, the majority of them hold a university degree (53.9%), are currently working (64.3%) and declared the presence of premenstrual syndrome at any level of severity (56.6%). A total of 155 out of 230 women had a laparoscopic confirmation of the presence of endometriosis lesions at the time of this survey, while in 62 (27.0%) the diagnosis was based on magnetic resonance imaging (MRI) and/or ultrasound (US) imaging techniques. Only 13 (5.7%) were diagnosed based on symptoms but not confirmed by MRI and/or US imaging. Finally, the mean time since diagnosis was 5.0 ± 5.3 years, and 68 (29.6%) had undergone at least two endometriosis surgeries.

### 3.1. Intensity of Chronic Fatigue and Pain in Spanish Women Diagnosed with Endometriosis

Self-reported severity of chronic fatigue and last week PP are summarized in Table 2. GM (±GSD) intensity of chronic fatigue was 5.9 ± 1.7 points, with almost half of the participating women reporting severe fatigue. Concerning last week PP intensity, GM (±GSD) was 5.0 ± 1.9. A total of 46.3% and 27.1% of the entire study population showed moderate and severe PP during the last week. Using multivariate linear regression modelling, a positive association was found between intensities of both chronic fatigue and last week PP scores after adjustment for potential confounders (Supplementary Table S1).

### 3.2. Psychosocial Impairments and Quality of Life in Spanish Women Diagnosed with Endometriosis

Table 3 summarizes the results from the descriptive analysis of each analyzed psychosocial dimension. Considering available cut-off scores (not shown in tables), anxiety was present in 169 out of 230 (73.4%) of the women, with 58 (25.2%) and 48 (20.9%) showing moderate and severe anxiety, respectively. Similarly, depression was detected in 111 (48.3%) of the women, with 45 (19.6%) and 19 (8.3%) showing moderate and severe depression, respectively. Additionally, pain catastrophizing thoughts were found in 108 (47.0%) of the participants, while poor sleep quality and sexual dysfunction were found in 187 (81.3%) and 174 (75.7%) of the participating women, respectively. Moreover, anger/hostility and happiness dimensions, assessed through the EVEA subscales, showed a GM (±GSD) of 12.2 ± 2.6 and 11.1 ± 2.3 points, respectively. GM (±GSD) MOS-SSS score was 73.7 ± 18.7 points, while for gastrointestinal problems, GM (±GSD) GIQLI score was 65.2 ± 1.4 points. Regarding QoL, GM (±GSD) EHP-30 score was 55.0 ± 1.7 points.

### 3.3. Contribution of Fatigue Intensity to Psychosocial Impairment in Spanish Women

**Figure 1:**
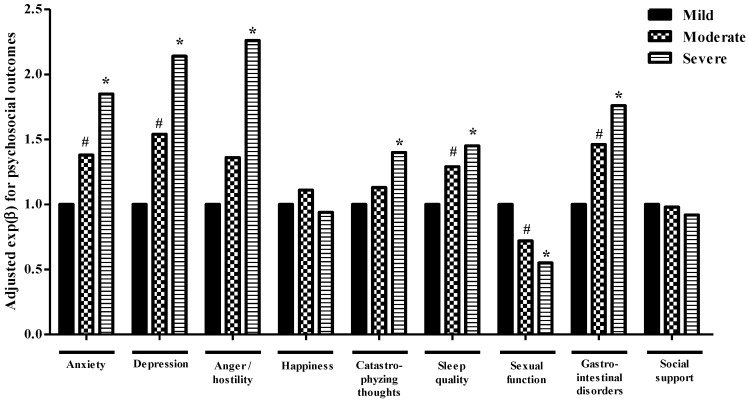
Influence of chronic fatigue on psychosocial status in Spanish women with endometriosis. Results from multivariate linear regression analyses adjusted for age (years), schooling, civil status, number of surgeries, type of diagnosis, time since diagnosis, number of children, premenstrual symptom severity and last week pelvic pain intensity. ^#^ *p*-value ≤ 0.05 between mild and moderate groups; * *p*-value ≤ 0.05 between mild and severe groups.

Results from the multivariate analyses assessing associations between self-perceived fatigue severity and psychosocial impairments are depicted in Figure 1. Results from the bivariate and multivariate analyses are summarized in Supplementary Table S2. After adjustment for potential confounders (sociodemographic and gynecological characteristics and intensity of PP during the last week), moderate and severe fatigue was found to be related to anxiety and depression, poorer sleep quality, poorer sexual functioning and less gastrointestinal quality of life in an intensity-dependent manner, while higher PCS and EVEA-anger/hostility scores were associated with severe fatigue. Moreover, multivariate logistic regression analyses that were run in parallel when cut-off points were available showed similar results (data not shown in tables). Sensitivity analyses stratified by endometriosis diagnosis yielded similar results.

### 3.4. Contribution of Pain, Fatigue and Psychosocial Impairment to Quality of Life in Spanish Women

Results from the multivariate linear regression analyses are summarized in Table 4. Severity of chronic fatigue and last week PP were associated with poorer QoL in an intensity-dependent manner. Moreover, anxiety, depression, anger/hostility and catastrophizing thoughts were associated with poorer QoL. Similarly, poorer gastrointestinal health, sexual function and sleep quality were also related to poorer QoL, although the latter showed a close to statistically significant association with QoL when models were further adjusted for intensity of PP during the last week (*p*-value 0.059).

**Figure 2:**
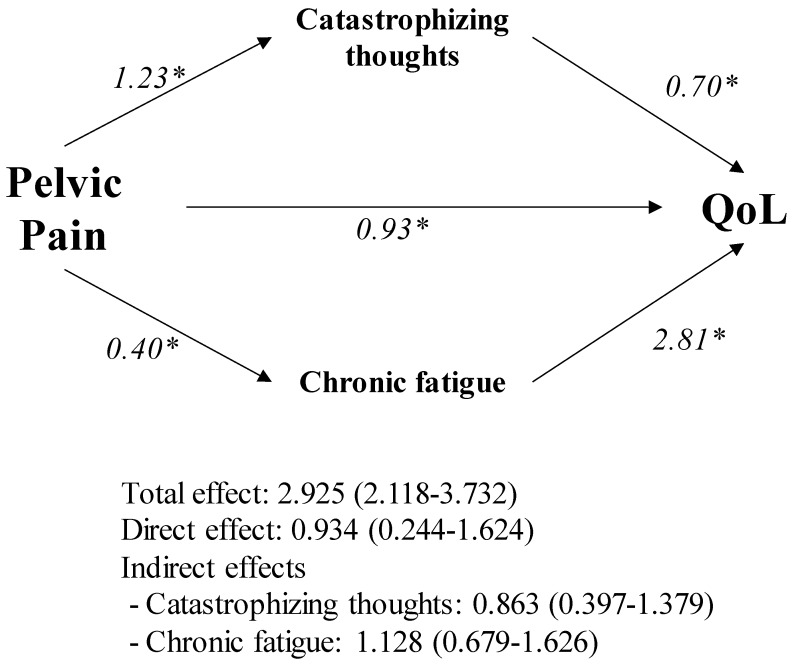
Mediating effect of chronic fatigue and catastrophizing thoughts on the association between pelvic pain and quality of life (QoL) in Spanish women with endometriosis. * Mediating effects were considered significant when zero was not located within the confidence intervals. Analyses were conducted with the macro PROCESS for SPSS.

Mediation effects of fatigue and psychosocial impairments on QoL were also accomplished (Figure 2). All chronic fatigue, gastrointestinal complaints, sexual function, anxiety, depression, anger/hostility, sleep quality and catastrophizing thoughts showed a mediating effect on the association between last week PP and QoL when assessed on an individual level (data not shown). However, when the combined mediating effect was evaluated, only chronic fatigue and catastrophizing thoughts revealed a statistically significant mediating effect on the association between last week PP and QoL (1.128 and 0.863, respectively; *p*-value < 0.05).

## 4. Discussion

To our knowledge, this study constitutes the first attempt to objectively evaluate levels of endometriosis-related fatigue in a comprehensive population, and to address its relevant contribution to the psychosocial status and QoL in Spanish women diagnosed with endometriosis. Moreover, our results suggest that endometriosis-related fatigue and catastrophizing thoughts also exert a mediating effect on the association between intensity of PP and poorer QoL in affected women, evidencing that these factors also need to be addressed within appropriate treatment approaches in women with endometriosis.

Previous studies have stated that women with endometriosis frequently report the presence of chronic fatigue [46], with authors defending the effect of endometriosis on its generation, independently from other symptoms of the disease [11]. Our study shows that 85.3% of the patients enrolled have moderate to severe fatigue. Our findings are in accordance with previous studies where affected women were asked if they felt fatigue. Hence, a total of 50.7% and 27.1% of women reported frequent and occasional fatigue, respectively [11]. Similarly, Surrey et al. [47] recently reported that 54–74% of affected women with moderate to severe pain reported experiencing fatigue. Although the underlying mechanisms are not still fully elucidated, it has been reported that the endometriotic lesions usually develop a complex and dynamic environment dominated by inflammatory, angiogenic, and endocrine signals [48]. Similarly, Suryawanshi et al. [49] reported that endometriotic lesions generate a specific immune microenvironment similar to a tumor-like inflammatory profile. Thus, in accordance with the positive correlation between inflammatory cytokines and fatigue shown in cancer patients [50], elevated cytokine levels found in endometriosis might be hypothesized to play a role in the development of fatigue symptomology in these affected women.

Regarding the relationship identified in this study between levels of fatigue experienced by women with endometriosis and severity of PP, it was not unexpected, as this association has been previously stated in different populations suffering different chronic conditions such as rheumatic diseases [51] or cancer [52]. In fact, both symptoms have been found to be related to an inflammatory microenvironment. Hence, studies from basic sciences evidenced that changes in immune surveillance and central sensitization were related to the pathophysiology of endometriosis [53]. Interestingly, a misbalance in estrogen levels, as widely reported in patients with endometriosis, may be the first responsible for the generation of an inflammatory microenvironment [48] that ultimately can lead to the development of not only PP [54] but also endometriosis-related fatigue. Moreover, the dysregulation of the hypothalamic–pituitary–adrenal (HPA) axis has been reported to contribute to the development of fatigue in chronic illness [55].

Our sample of patients show high levels of psychosocial impairments such as anxiety, depression, poor sleep quality or sexual dysfunction. In this respect, several studies have stated the association between endometriosis and psychological impairments, with depression and anxiety as the most common disorders related to endometriosis, deeply affecting the QoL of these women [6,56,57,58,59]. Interestingly, our results suggest an association of chronic fatigue and psychosocial factors, as reported in another multicenter study by Ramin-Wright et al. [11] that comprised 554 women with endometriosis, where fatigue was associated with insomnia and depression among other factors. In this respect, a previous review stated the influence of chronic fatigue on different inflammatory conditions and the possible association between inflammation, pain and depression [51,60]. Moreover, in addition to poorer sleep quality and depression, our study suggests for the first time that the presence of chronic fatigue is associated with higher levels of anxiety and anger/hostility, as well as poorer sexual function. In accordance with our results, it has been reported that fatigue was associated with poorer sexual functioning in women with chronic conditions such as breast cancer [61] or multiple sclerosis [62]. In this regard, fitness level, crucially related to the presence of chronic fatigue, has been recently identified as a strong predictor of sexual function in middle-aged adult women [63]. Although the molecular links between chronic fatigue and psychosocial impairments remain unclear, it has been suggested that the HPA axis might be behind this cluster of symptoms typically observed in cancer patients [64]. Interestingly, an aberrant HPA response has been reported in women with endometriosis [65]. Moreover, social factors might also contribute to the development of fatigue. Hence, in a different subset of patients, it has been reported that social support, through promoting self-confidence and rational thoughts, may have an impact on the reinforcement of the immunity system, and in turn, on the reduction of fatigue levels [66]. Care practitioners and clinicians’ perception of women’s experiences of endometriosis [67] and low-value healthcare [68] might also contribute to the endometriosis-related burden of symptoms. Additionally, diagnosis delay, infertility and worries related to low work productivity or job loss, in addition to depressive symptoms or disturbed sleep, might also negatively impact on energy and vitality [69,70,71], revealing the complex inter-relationships between physiological and psychosocial factors in women with endometriosis.

Regarding the interrelationship between intensity of PP, chronic fatigue and psychosocial impairments, our findings indicate that chronic fatigue and catastrophizing thoughts may mediate the association between last week PP intensity and QoL. A similar relationship was described in a previous work showing an association between pain and psychological stress with a worsening of the QoL in women living with endometriosis [72]. In this study, we have added for the first time the contribution of chronic fatigue, in addition to psychosocial distress, on QoL impairment in women with endometriosis. Taken together, these studies would support the idea that pain is associated with chronic fatigue and negative emotions [73,74], that in turn could affect QoL in women with endometriosis. Therefore, our data supports the necessity of multimodal treatments that address fatigue and psychosocial distress in addition to PP intensity in order to improve QoL in women with endometriosis, in line with previous suggestions [11,72]. Thus, besides medical therapy [47], physical and psychological interventions might be beneficial in endometriosis treatment, as evidenced for a variety of chronic illnesses in women [75,76,77,78]. More attention should be paid to non-pharmacological approaches to manage the symptom burden of this silenced female disease.

Regarding limitations, study population might not be fully representative of Spanish women with endometriosis. In this regard, although we have included affected women from all Spanish regions, the presence of a selection bias is plausible, given that participants might have a different symptom burden than non-participants. In this regard, it has been reported that outcomes related to QoL are influenced by recruitment strategy [79]. Secondly, this study has a cross-sectional design that does not allow the assessment of the causal relation between studied variables. The absence of a control group also limited understanding of the differential impact of this symptom on lives of women with and without endometriosis. Nevertheless, in a case-control study comprised of 25 women with endometriosis and 25 healthy controls, we have recently reported that mean fatigue score was 2.9 ± 2.0 among controls and 5.3 ± 2.3 among women with endometriosis [80]. In fact, a large majority of healthy women had mild fatigue and none of them had severe fatigue. Contrarily, the majority of women with endometriosis (72.0%) had moderate-severe fatigue. Moreover, the information retrieved in the present study was obtained from self-administered questionnaires and, therefore, a risk of information bias could also exist. However, the use of validated scales for this assessment may counteract this information bias. Finally, we have no information about medication taken by the women during the study, although all participants reported to be adhering to the prescribed medical treatment. However, it is possible that fatigue in endometriosis could be partially attributed to side effects from medication [46]. In addition, contraceptive hormonal therapy is usually prescribed to many women with endometriosis, and its use has been previously associated with different depression symptoms [81].

## 5. Conclusions

This work provides preliminary evidence of the relevance of chronic fatigue for the psychosocial status and the QoL of women living with endometriosis. We consider that it has important implications for the evaluation and treatment of this population, as the main goal of their management is usually to ameliorate symptoms and to improve general QoL. The habitual treatment of the disease is focused on classic symptoms such as pain or infertility [1,82], but our findings also support the importance of addressing fatigue when treating patients with endometriosis, highlighting the necessity for an interdisciplinary management of the disease. Thus, our results warrant future studies that assess the effectiveness of multidisciplinary approaches (i.e., physical and psychological rehabilitation interventions, in addition to medical therapy) for symptom management.

## Figures and Tables

**Table 1:** Baseline characteristics of the study population (N = 230).

	N (%)		N (%)
***Sociodemographic characteristics***		***Gynecological characteristics***	
**Age** (years)*	36.4 ± 1.2	**N° children**	
**Schooling**		*None*	162 (70.4)
*Primary/secondary studies*	43 (18.7)	*1*	40 (17.4)
*Vocational training*	63 (27.4)	*2*	23 (10.0)
*University studies*	124 (53.9)	*3*	5 (2.2)
**Civil status**		**PMS severity**	
*Single/Divorced*	112 (48.7)	*None*	100 (43.5)
*Married*	118 (51.3)	*Mild*	14 (6.1)
**Working outside home**		*Moderate*	77 (33.5)
*No*	26 (11.3)	*Severe*	39 (17.0)
*No, sick leave*	30 (13.0)	**Endometriosis diagnosis**	
*No, loss due to endometriosis*	26 (11.3)	*Laparoscopy*	155 (67.4)
*Yes*	148 (64.3)	*MRI and/or US*	62 (27.0)
		*Based on symptoms*	13 (5.7)
		**Time since endometriosis diagnosis** (years) *	2.4 ± 4.6
		**N° surgeries**	
		*None*	75 (32.6)
		*1 surgery*	87 (37.8)
		*2 surgeries*	40 (17.4)
		*3 or more surgeries*	28 (12.2)

* Geometric mean ± geometric standard deviation; PMS: premenstrual symptoms; MRI: magnetic resonance imaging; US: ultrasound.

**Table 2:** Intensity of chronic fatigue and pelvic pain in women with endometriosis.

	N
**Chronic fatigue**	5.9 ± 1.7 *
*Mild (<4)*	34 (14.8)
*Moderate (4–7)*	82 (35.7)
*Severe (>7)*	114 (49.6)
**Last week pelvic pain intensity**	5.0 ± 1.9 *
*Mild (<4)*	64 (26.7)
*Moderate (4–7)*	111 (46.3)
*Severe (>7)*	65 (27.1)

* Geometric mean ± geometric standard deviation.

**Table 3:** Psychosocial status and quality of life in women with endometriosis.

	GM	GSD	Min.	Percentiles			Max.
				25	50	75	
**Quality of life**							
*Total score*	55.0	1.7	1.7	47.2	62.5	73.5	96.6
**Gastrointestinal quality of life**							
*Total score*	65.2	1.4	11.0	56.8	70.0	82.0	116.0
**Sexual function**							
*Total score*	14.1	2.3	2.0	9.8	19.7	25.8	36.0
**Mental health**							
*Anxiety*	9.4	1.7	1.0	7.0	10.0	13.3	20.0
*Depression*	6.6	1.9	0.0	4.0	7.0	11.0	21.0
**Pain catastrophizing scale**							
*Total score*	23.4	2.0	0.0	17.0	28.0	39.0	52.0
**Sleep quality**							
*Total score*	9.2	1.7	1.0	6.8	10.0	14.0	21.0
**Scale for Mood Assessment**							
*Anger hostility*	12.2	2.6	0.0	4.0	13.0	26.0	40.0
*Happiness*	11.1	2.3	0.0	5.0	13.0	21.0	38.0
**Medical Outcomes Study-Social Support Survey**							
*Total score*	70.8	1.4	23.0	59.8	76.5	90.0	95.0

GM: geometric mean; GSD: geometric standard deviation.

**Table 4:** Relationship between psychosocial impairments and quality of life in women with endometriosis. Linear regression analyses.

	Bivariate Analysis				Adjusted Analysis *				Adjusted Analysis **			
	Exp (β)	95%CI		*p*-Value	Exp (β)	95%CI		*p*-Value	Exp (β)	95%CI		*p*-Value
**Self-perceived pelvic pain severity (NRS)**												
*NRS = Moderate*	1.39	1.20	1.62	<0.001	1.28	1.09	1.50	0.003	-	-	-	-
*NRS = Severe*	1.50	1.26	1.78	<0.001	1.39	1.15	1.68	0.001	-	-	-	-
**Chronic fatigue (PFS)**												
*PFS = Moderate*	1.41	1.17	1.71	<0.001	1.42	1.18	1.71	<0.001	1.37	1.13	1.65	0.001
*PFS = Severe*	1.74	1.45	2.08	<0.001	1.67	1.39	2.01	<0.001	1.56	1.28	1.91	<0.001
**Gastrointestinal quality of life (GIQLI)**												
*Total score*	1.96	1.66	2.32	<0.001	1.83	1.51	2.23	<0.001	1.83	1.51	2.23	<0.001
**Sexual function (FSFI)**												
*Total score*	0.87	0.80	0.94	<0.001	0.89	0.82	0.96	0.004	0.91	0.84	0.99	0.022
**Mental health (HADS)**												
*Anxiety*	1.43	1.27	1.61	<0.001	1.33	1.18	1.50	<0.001	1.29	1.15	1.46	<0.001
*Depression*	1.29	1.18	1.41	<0.001	1.25	1.13	1.37	<0.001	1.21	1.09	1.34	<0.001
**Pain catastrophizing scale (PCS)**												
*Total score*	1.52	1.41	1.63	<0.001	1.49	1.38	1.61	<0.001	1.47	1.35	1.59	<0.001
**Sleep quality (PSQI)**												
*Total score*	1.30	1.16	1.46	<0.001	1.21	1.06	1.38	0.005	1.14	0.99	1.31	0.059
**Scale for Mood Assessment (EVEA)**												
*Anger hostility*	1.13	1.06	1.22	<0.001	1.09	1.02	1.17	0.012	1.08	1.01	1.15	0.031
*Happiness*	1.02	0.94	1.10	0.674	1.01	0.94	1.09	0.782	1.04	0.96	1.12	0.346
**Medical Outcomes Study-Social Support Survey (MOS-SSS)**												
*Total score*	0.73	0.59	0.91	0.005	0.79	0.63	1.00	0.048	0.83	0.66	1.03	0.096

NRS: numeric rating scale; PFS: Piper Fatigue Scale; GIQLI: Gastrointestinal Quality of Life Index; FSFI: Female Sexual Function Scale; HADS: Hospital Anxiety and Depression Scale; PCS: Pain Catastrophizing Scale; PSQI: Pittsburgh Sleep Quality Index; EVEA: Scale for Mood Assessment; MOS-SSS: Medical Outcomes Study-Social Support Survey. * Adjusted for age (yrs), schooling, civil status, number of surgeries, type of diagnosis, time since diagnosis, number of children and PMS severity; ** Additionally adjusted for intensity of pelvic pain during last week.

## Supplementary Materials

The following are available online at https://www.mdpi.com/1660-4601/17/11/3831/s1, Table S1. Relationship between intensity of chronic pelvic pain and chronic fatigue; Table S2. Relationship between intensity of chronic fatigue, psychosocial status and quality of life in women with endometriosis.
