# Supplementary material for: Contribution of Chronic Fatigue to Psychosocial Status and Quality of Life in Spanish Women Diagnosed with Endometriosis
Source: Int J Environ Res Public Health. 2020 May 28;17(11):3831. doi: 10.3390/ijerph17113831 (PMC7312817; doi:10.3390/ijerph17113831)
Supplement: Supplementary file 1 [file ijerph-17-03831-s001.pdf]

Table S1. Relationship between intensity of chronic pelvic pain and chronic fatigue.

	Bivariate analysis									
	lwPP == *Moderate*					lwPP == *Severe*				
	**β**	**Exp (β)**	**95%CI**		***p*-value**	**β**	**Exp (β)**	**95%CI**		***p*-value**
**Chronic fatigue**										
Total score	0.37	1.45	1.25	1.69	<0.001	0,58	1.79	1.51	2.12	<0.001
Behavioral	0.45	1.57	1.32	1.87	<0.001	0.72	2.05	1.69	2.49	<0.001
Affective	0.34	1.40	1.17	1.68	<0.001	0.59	1.80	1.48	2.20	<0.001
Sensory	0.37	1.44	1.22	1.70	<0.001	0.54	1.72	1.43	2.06	<0.001
Cognitive	0.40	1.49	1.27	1.76	<0.001	0.58	1.79	1.50	2.15	<0.001

	Adjusted analysis*									
	lwPP == *Moderate*					lwPP == *Severe*				
	**β**	**Exp (β)**	**95%CI**		***p*-value**	**β**	**Exp (β)**	**95%CI**		***p*-value**
**Chronic fatigue**										
Total score	0.40	1.49	1.27	1.75	<0.001	0.56	1.75	1.44	2.12	<0.001
Behavioral	0.46	1.58	1.31	1.90	<0.001	0.66	1.94	1.56	2.42	<0.001
Affective	0.39	1.47	1.21	1.79	<0.001	0.56	1.76	1.39	2.22	<0.001
Sensory	0.40	1.49	1.25	1.79	<0.001	0.54	1.71	1.38	2.12	<0.001
Cognitive	0.42	1.53	1.29	1.82	<0.001	0.57	1.77	1.44	2.17	<0.001

lwPP: average of last week pelvic pain intensity *Adjusted for age (yrs), schooling, civil status, number of surgeries, type of diagnosis, time since diagnosis, number of children and PMS severity

Table S2. Relationship between intensity of chronic fatigue, psychosocial status and quality of life in women with endometriosis.

	Adjusted analyses*									
	PFS == *Moderate*					*PFS* == *Severe*				
	**β**	**Exp (β)**	**95%CI**		***p*-value**	**β**	**Exp (β)**	**95%CI**		***p*-value**
**Quality of life (EHP-30)**										
Total score	0.35	1.42	1.18	1.71	<0.001	0.51	1.67	1.39	2.01	<0.001
**Gastrointestinal quality of life (GIQLI)**										
Total score	0.41	1.50	1.36	1.67	<0.001	0.62	1.86	1.68	2.05	<0.001
**Sexual function (FSFI)**										
Total score	−0.37	0.69	0.51	0.94	0.019	−0,68	0.51	0.38	0.69	<0.001
**Mental health (HADS)**										
Anxiety	0.33	1.39	1.14	1.69	0.001	0.62	1.86	1.53	2.25	<0.001
Depression	0.47	1.60	1.27	2.01	<0.001	0.87	2.38	1.90	2.99	<0.001
**Pain catastrophyzing scale (PCS)**										
Total score	0.17	1.18	0.90	1.54	0.220	0.44	1.55	1.19	2.03	0.001
**Sleep quality (PSQI)**										
Total score	0.31	1.36	1.13	1.63	0.001	0.48	1.62	1.34	1.94	<0.001
**Scale for Mood Assessment (EVEA)**										
Anger hostility	0.32	1.37	0.89	2.12	0.151	0.83	2.30	1.50	3.52	<0.001
Happiness	0.04	1.05	0.74	1.48	0.804	−0.22	0.81	0.57	1.14	0.222
**Medical Outcomes Study-Social Support Survey (MOS-SSS)**										
Total score	−0.02	0.98	0.87	1.10	0.686	−0,10	0.91	0.81	1.01	0.087

	Adjusted analyses**									
	PFS == *Moderate*					*PFS* == *Severe*				
	**β**	**Exp (β)**	**95%CI**		***p*-value**	**β**	**Exp (β)**	**95%CI**		***p*-value**
**Quality of life (EHP-30)**										
Total score	0.31	1.37	1.13	1.65	0.001	0.45	1.56	1.28	1.91	<0.001
**Gastrointestinal quality of life (GIQLI)**										
Total score	0.38	1.46	1.32	1.62	<0.001	0.56	1.76	1.57	1.96	<0.001
**Sexual function (FSFI)**										
Total score	–0.33	0.72	0.53	0.98	0.037	–0.61	0.55	0.39	0.76	<0.001
**Mental health (HADS)**										
Anxiety	0.33	1.38	1.13	1.69	0.002	0.61	1.85	1.50	2.28	<0.001
Depression	0.43	1.54	1.22	1.94	<0.001	0.76	2.14	1.68	2.72	<0.001
**Pain catastrophyzing scale (PCS)**										
Total score	0.12	1.13	0.86	1.49	0.373	0.34	1.40	1.05	1.86	0.021
**Sleep quality (PSQI)**										
Total score	0.25	1.29	1.07	1.55	0.008	0.37	1.45	1.19	1.76	<0.001
**Scale for Mood Assessment (EVEA)**										
Anger hostility	0.31	1.36	0.87	2.13	0.174	0.82	2.26	1.42	3.59	0.001
Happiness	0.10	1.11	0.78	1.58	0.570	−0.06	0.94	0.65	1.37	0.757
**Medical Outcomes Study-Social Support Survey (MOS-SSS)**										
Total score	−0.02	0.98	0.87	1.11	0.795	−0.08	0.92	0.82	1.04	0.204

PFS: Piper Fatigue Scale; GIQLI: Gastrointestinal Quality of Life Index; FSFI: Female Sexual Function Scale; HADS: Hospital Anxiety and Depression Scale; PCS: Pain Catastrophizing Scale; PSQI: Pittsburgh Sleep Quality Index; EVEA: Scale for Mood Assessment; MOS-SSS: Medical Outcomes Study-Social Support Survey. *Adjusted for age (yrs), schooling, civil status, number of surgeries, type of diagnosis, time since diagnosis, number of children and PMS severity; **Additionally adjusted for average of last week pelvic pain intensity.
